# Charge-Coupled Frequency Response Multispectral Inversion Network-Based Detection Method of Oil Contamination on Airport Runway

**DOI:** 10.3390/s24123716

**Published:** 2024-06-07

**Authors:** Shuanfeng Zhao, Zhijian Luo, Li Wang, Xiaoyu Li, Zhizhong Xing

**Affiliations:** 1 College of Mechanical Engineering, Xi’an University of Science and Technology, Xi’an 710054, China; zhijianluo16037@163.com (Z.L.); wl199710042024@163.com (L.W.); xiaoyuli0403@163.com (X.L.); 2 School of Rehabilitation, Kunming Medical University, Kunming 650500, China; zzxing64@163.com

**Keywords:** spectral reconstruction, multispectral images, convolutional neural networks, target detection, oil contamination

## Abstract

Aircraft failures can result in the leakage of fuel, hydraulic oil, or other lubricants onto the runway during landing or taxiing. Damage to fuel tanks or oil lines during hard landings or accidents can also contribute to these spills. Further, improper maintenance or operational errors may leave oil traces on the runway before take-off or after landing. Identifying oil spills in airport runway videos is crucial to flight safety and accident investigation. Advanced image processing techniques can overcome the limitations of conventional RGB-based detection, which struggles to differentiate between oil spills and sewage due to similar coloration; given that oil and sewage have distinct spectral absorption patterns, precise detection can be performed based on multispectral images. In this study, we developed a method for spectrally enhancing RGB images of oil spills on airport runways to generate HSI images, facilitating oil spill detection in conventional RGB imagery. To this end, we employed the MST++ spectral reconstruction network model to effectively reconstruct RGB images into multispectral images, yielding improved accuracy in oil detection compared with other models. Additionally, we utilized the Fast R-CNN oil spill detection model, resulting in a 5% increase in Intersection over Union (IOU) for HSI images. Moreover, compared with RGB images, this approach significantly enhanced detection accuracy and completeness by 25.3% and 26.5%, respectively. These findings clearly demonstrate the superior precision and accuracy of HSI images based on spectral reconstruction in oil spill detection compared with traditional RGB images. With the spectral reconstruction technique, we can effectively make use of the spectral information inherent in oil spills, thereby enhancing detection accuracy. Future research could delve deeper into optimization techniques and conduct extensive validation in real airport environments. In conclusion, this spectral reconstruction-based technique for detecting oil spills on airport runways offers a novel and efficient approach that upholds both efficacy and accuracy. Its wide-scale implementation in airport operations holds great potential for improving aviation safety and environmental protection.

## 1. Introduction

When an airport accident occurs, it is extremely important to be able to reconstruct the accident in a timely manner. In this process, the full use of video information during take-off or landing is of great importance in analyzing the causes of accidents and preventing potential ones. According to historical data and research, the processes of aircraft take-off and landing are phases at high risk of in-flight accidents. Identifying oil spills on the runway is one of the key factors in analyzing the causes of these events [1]. In some cases, aircraft may leave oil stains on the runway before take-off or after landing due to improper maintenance or operational errors, representing a potential factor contributing to an accident. The main sources of oil spills in airports are fuel spills from aircraft parking and landing and fuel system leaks from ground service vehicles. Although runway video information can provide a visual record of, for example, oil spills, there are still some challenges to its practical use, such as the similarity in color between oil and sewage, as well as other factors. However, oil and sewage have different absorption patterns in spectra of different wavelengths, so they present discernible differences in multispectral images, allowing for their accurate detection [2]. According to International Civil Aviation Organization (ICAO) Circular 355, the presence of deposits such as oil and mud between aircraft tires and the runway poses a significant hazard to aircraft during take-off and landing and a serious threat to public safety. Based on the above, in this study, we designed a new method to generate multispectral images by spectrally enhancing traditional RGB images of oil spills on airport runways for detection. This method can be applied not only to reconstruct airport accidents but also to keep airport runways safe. By using spectral enhancement techniques, we are able to accurately identify and locate oil spills in images so that timely cleaning and maintenance measures can be taken to ensure the safety of the aircraft landing and take-off processes. The application of this method could improve accuracy in airport accident reconstruction and help to ensure runway safety, providing a higher overall level of air transport safety [3]. By making full use of video information relative to take-off and landing and obtaining accurate data on oil spills, we will be able to better analyze the causes of accidents and identify potential accident factors, thus improving flight safety.

Currently, several researchers have made noteworthy contributions to oil spill detection. Zahra Ghorbani and colleagues [4] successfully utilized multi-class convolutional neural networks for offshore oil spill detection, while Mohammed S. Ozigis and others [5] employed multispectral images along with fuzzy forest and random forest methods to detect oil spills. In the context of airport runway oil spill detection, the prevalent methods primarily rely on RGB images captured with RGB cameras. However, it is important to note that these devices solely collect spectral information within the red, green, and blue bands; consequently, they fail to directly capture the specific spectral characteristics relevant to oil spill detection, thereby limiting the precise detection and differentiation of oil spills. Moreover, in runway oil spill detection, environmental factors introduce further interference in RGB images, such as the presence of sewage and other forms of spillage, as well as the potential similarity in coloration between sewage and oil. Consequently, environmental factors can impede accurate detection when relying solely on RGB images. In contrast, multispectral images offer a wealth of spectral information [6], enhanced differentiation capabilities [7], and improved quantitative analysis abilities [8]. They exhibit higher levels of detection accuracy and reliability. Multispectral images have found extensive applications in diverse fields, including agriculture [9], food detection [10], plant pathology analysis [11], material identification [12], and even oil spill detection on the sea surface [13]. Despite the wide-ranging applications of multispectral imagery, the direct implementation of multispectral cameras for detecting oil spills on airport runways presents certain drawbacks. Firstly, acquiring multispectral images can be time-consuming, and the equipment required for multispectral cameras is typically bulkier and less portable. Given that swift deployment and detection of foreign objects are essential in airport environments, the direct use of multispectral cameras for airport runway oil spill detection is constrained. Moreover, if an accident occurs at an airport, it is difficult to obtain oil information from RGB images, and so is tracing the accident back to its origin; however, because of the specific spectral characteristics of oil, the integration of multispectral and RGB imaging might offer a solution [14]. In light of these considerations, in this study, we established a method whereby RGB images of airport runway oil spills, acquired with RGB cameras, undergo spectral amplification to generate multispectral images specifically for oil spill detection. This approach presents superior detection accuracy and efficiency, thereby enabling the use of ordinary RGB cameras for airport runway oil spill detection.

Airport runway oil spill detection plays a crucial role in ensuring aviation safety and facilitating prompt accident investigations. Traditional methods based on RGB image analysis often struggle to accurately distinguish between oil spills and water stains due to their similar visual appearances. To address this limitation, we propose a novel airport runway oil spill detection method that leverages spectral reconstruction techniques to enhance the discriminative power of the captured images.

The proposed method consists of the following key steps: Firstly, RGB image data of oil spills on airport runways are acquired by using a camera, and the latter’s spectral response curve is obtained. Subsequently, a spectral reconstruction dataset is generated by utilizing the response curve and is then used to train a spectral reconstruction network model. Next, the acquired RGB images are reconstructed into multispectral images, and oil spill detection is performed on both the original and reconstructed images with the Fast R-CNN model. Finally, evaluation metrics such as Intersection over Union (IoU) are employed to assess the differences in detection performance before and after reconstruction, validating the effectiveness and practicality of the proposed method.

The main contributions of this study in the field of airport runway oil spill detection are as follows:

1. An innovative airport runway oil spill detection method that effectively overcomes the limitations of traditional RGB image detection in distinguishing oil spills from water stains by spectrally reconstructing RGB images into multispectral images, thereby achieving high-precision oil spill detection, is proposed.
2. The high-performance MST++ spectral reconstruction network model is adopted in combination with the Fast R-CNN oil spill detection model, resulting in significant improvements in accuracy and completeness in oil spill detection. Compared with directly utilizing RGB images, the proposed method increases Intersection over Union (IOU) by 5%, while detection accuracy and completeness are enhanced by 25.3% and 26.5%, respectively. Moreover, successful detection is achieved in various scenarios.
3. The proposed method offers higher practicality and convenience. By capturing images with ordinary RGB cameras and then generating multispectral images through spectral reconstruction for detection, the method significantly improves detection accuracy while greatly enhancing equipment portability, making it more suitable for the practical application requirements of airport runways.

## 2. Materials and Methods

### 2.1. Preparation of the Dataset

#### 2.1.1. Characteristics of Oil Contamination

**Figure 1:**
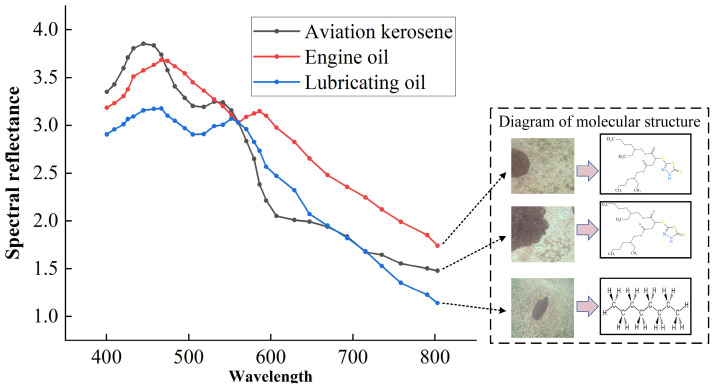
Oil characteristics.

Oil spillage on airport pavement is a prevalent issue and predominantly comprises aviation kerosene, motor oil, lubricants, and similar substances. These oil contaminants often exhibit strikingly similar colors when captured in RGB images, posing challenges for accurate detection based on traditional visual inspection and detection methods and effective cleanup. However, it is worth noting that different types of oil spills exhibit varying levels of reflectivity. This fundamental insight forms the theoretical basis for leveraging multispectral images in the detection of oil spills on airport pavement. In a study conducted by Lai, X. [15], the authors conducted extensive testing and analysis on oil spills including paraffin, motor oil, and lubricating oil in oceanic environments. The results revealed discernible differences in the reflectance properties of various oil spill types, as depicted in Figure 1 below.

Moreover, it is worth noting that the reflectivity of different oil types on the road surface exhibits variations, presenting a promising avenue for leveraging multispectral imagery in the detection of oil spills on airport runways. Consequently, the utilization of multispectral imagery holds the potential to enhance the accuracy and dependability of identification and detection processes in the context of airport runway oil spillage. This breakthrough opens up new possibilities for improving efficiency in oil spill detection in airports, thus paving the way for enhanced environmental stewardship in aviation settings.

#### 2.1.2. Data Collection

**Figure 2:**
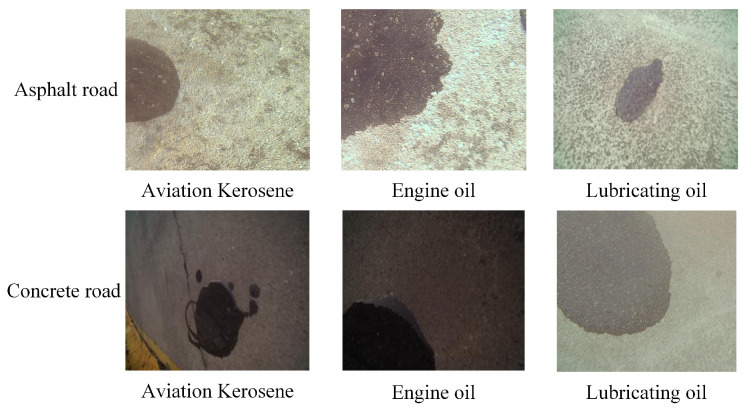
Partial dataset.

The dataset utilized in this study was collected from an airport located in Shanxi, China, and encompassed a diverse range of samples representing airport runway oil spills, including instances of aviation paraffin, motor oil, and lubricants. To optimize the reconstruction speed and accuracy of RGB images depicting oil spills on airport pavement, the data acquisition process was specifically conducted during periods of clear weather conditions. To account for the intricate nature of airport runway oil spillage, the dataset was further enriched by incorporating a mixture of aviation paraffin, motor oil, and lubricating oil, thereby encompassing a comprehensive representation of oil spill types commonly encountered on airport runways. Furthermore, given the varied composition of airport pavement, separate datasets were created for both asphalt and concrete surfaces, ensuring a comprehensive coverage of diverse pavement materials. Figure 2 provides a visual representation of some selected samples from the dataset, offering a glimpse into its richness and diversity.

#### 2.1.3. Processing of Datasets

In general, if the light intensity is high, the photos of a dataset may be distorted, which may affect the reconstruction results. To ensure the reliability of a dataset, two reference colors (white and dark) can be used to calibrate the multispectral data according to Equation (1), with the aim of eliminating the effect of lighting conditions on the data to make the latter comparable and consistent under different conditions.

(1) $$I_{c}=\frac{I_{r}-I_{d}}{I_{w}-I_{d}}$$

In this study, the collected images of the dataset were 1280 × 1300 pixels, but when they were used as input for spectral reconstruction, the network model reported errors due to the large image size. To reduce the load on the network, we cropped the acquired dataset images to 1000 × 1000 pixels. By doing so, the input requirements of the network model were met, and the computational load was reduced, improving the operational stability of the reconstruction network.

### 2.2. Spectral Reconstruction Methods

Multispectral imaging, characterized by its broader bandwidth and reduced susceptibility to visible light interference [16], presents a remarkable opportunity for target detection due to its ability to capture richer spectral information. Consequently, multispectral imaging holds great promise for the detection of contaminants in airports, including oil spills on runways. However, it is important to acknowledge that the adoption of multispectral cameras poses certain limitations. These cameras tend to be expensive and less portable, making them unsuitable for the swift and efficient detection of foreign objects in airport environments. To overcome these challenges, in this study, we designed a novel approach that involves reconstructing multispectral images from a single RGB image, which can then be applied to the detection of oil spills on airport runways. This method enables the utilization of existing RGB cameras, offering a cost-effective and practical solution for enhancing oil spill detection without the need for specialized multispectral equipment.

#### 2.2.1. Sampling Imaging Principle

An RGB image is an image containing three bands. An RGB image is obtained by combining red, green, and blue colors of light to obtain other colors [17]. When the light source is daylight, the spectrum is continuous, and the output of the RGB image is given by the following Equation (2):

(2) $$p_{k}=\int_{\Omega }o\left(\lambda \right)c_{k}\left(\lambda \right)d\lambda$$

where k denotes the R, G, and B channels; λ denotes the wavelength; and o is the incident light.

An RGB camera is an imaging device that records images by the optical imaging principle, which transmits the target image path through an optical lens and receives the target image through a charge coupler [18]. The light source emits electromagnetic waves of a certain wavelength, and different objects have different reflectivity to electromagnetic waves. When the light source irradiates the target object, the reflection spectrum of the surface of the target object is captured by the camera and converted by the imaging device of the camera to obtain the RGB image. Without considering the influencing factors, the process of forming the RGB image can be expressed by the following Equation (3):

(3) $$I=\int E\left(\lambda \right)S\left(\lambda \right)CSS\left(\lambda \right)d\lambda$$

where $E\left(\lambda \right)$ is the spectral energy distribution of the light source, $S\left(\lambda \right)$ is the spectral reflectance of the object, CSS is the spectral response function of the camera, and λ is the wavelength of the electromagnetic wave, which typically ranges from 400 nm to 700 nm of visible light.

Mathematically, the imaging process of the RGB camera can be regarded as the product of the spectra of the object surface and the spectral response function of the RGB camera [19]. And the multiplication within Equation (3) is the spectral value of the object surface, so the following Equation (4) can be obtained:

(4) $$I=\int_{\Omega }HSI\left(n_{\lambda }\right)\times CSS\left(n_{\lambda }\right)d\lambda$$

where HSI is the object surface spectrum and CSS is the spectral response function of the RGB camera. Using Equation (4) above, it is clear that the most important thing to carry out when performing spectral reconstruction is to obtain the spectral response curve of the RGB camera.

#### 2.2.2. Acquisition of Camera Spectral Response Curves

When imaging with an RGB camera, a number of factors affect the camera’s spectral response curve, such as white balance (AWB) and exposure time [20]. These factors in turn affect the subsequent training of the network. In particular, white balance (AWB) affects the captured RGB values, leading to uncertainty in the mapping between the RGB values and the corresponding spectra. Therefore, during the calibration process, we set the RGB gain to 1:1:1 and fixed the exposure time of the camera.

**Figure 3:**
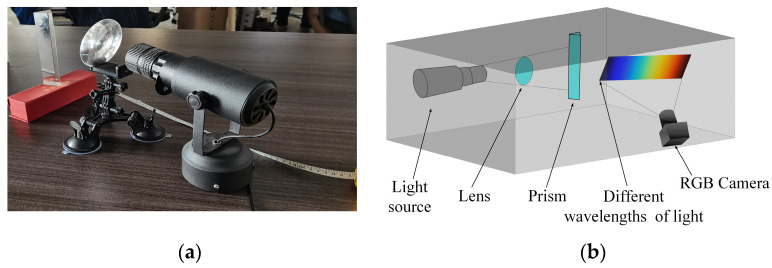
Spectral response curve acquisition platform. (**a**) Component display diagram of the platform. (**b**) Schematic diagram of experimental collection process.

The experimental system platform developed in this study is depicted in Figure 3, with Figure 3a showcasing the components and arrangement. The platform comprised essential elements such as a standardized light source, a convex lens, a prism, and an RGB camera, which collectively facilitate the acquisition of spectral images. The experimental process for acquiring these spectral images is illustrated in Figure 3b. Notably, the spectral acquisition procedure necessitates a controlled environment within a dark room to minimize external light interference and ensure accurate and reliable results. Meticulously maintaining a dark room environment during the spectral acquisition process was crucial to the success of this study.

**Figure 4:**
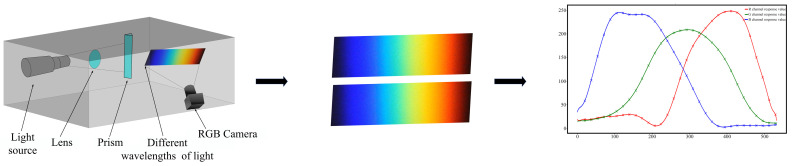
Spectral response curve acquisition. The various colour curves displayed in the graph represent the differing wavelength response values of the red, green and blue RGB channels, respectively.

To establish the color bar for the CCD camera under varying light intensities, an experimental platform was meticulously constructed. Each color block on the bar corresponded to the reflection or transmission characteristics of light at a particular wavelength. Based on precise measurements, the color bars were obtained, and the spectral data were subsequently transformed into a standardized spectral response curve. This process involved carefully processing and curve-fitting the spectral data, resulting in the derivation of the CCD camera’s spectral response curve, graphically represented in Figure 4, providing a comprehensive visual representation of the camera’s sensitivity to different wavelengths of light. Such a curve assisted in accurately interpreting and analyzing the acquired spectral images during the subsequent stages of this study.

### 2.3. Spectral Reconstruction and Runway Oil Detection Framework

**Figure 5:**
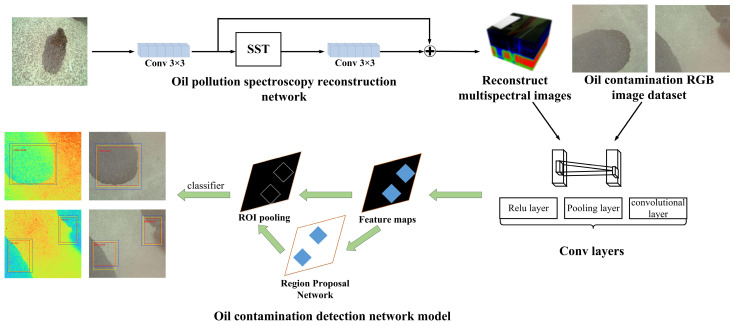
Schematic diagram of oil spectral reconstruction and oil detection network architecture.

The overall framework of this research study is depicted in Figure 5, providing a comprehensive overview of the methodology. The initial step involved acquiring the spectral response curve of the RGB camera employed in this investigation, as determined through the aforementioned experiments. Subsequently, the oil spill spectral reconstruction network model was trained on the original RGB data. In this process, the original RGB image of an oil spill is inputted into the oil spill spectral reconstruction network, resulting in the generation of a reconstructed oil spill hyperspectral imaging (HSI) image. Then, both the reconstructed and original images are utilized as inputs for the oil spill detection network. By comparing the outcomes of the respective tests, the final detection results are obtained. This comprehensive framework enabled the evaluation and comparison of detection performance between the reconstructed HSI image and the conventional RGB image, thereby providing valuable insights into the efficacy of the proposed spectral reconstruction method for airport runway oil spill detection.

#### 2.3.1. Spectral Reconstruction Neural Network

The main difficulty in reconstructing multispectral images from RGB images is the loss of information. Traditional spectral reconstruction methods, such as Principal Component Analysis (PCA) and Non-Negative Matrix Compression (NMF) [21], can downscale and compress spectral data but present the limitations of being sensitive to noise, being limited by linearity assumptions, and having low model complexity. For certain datasets, such as oil spills on airport runways, the performance of traditional methods may not be satisfactory.

In recent years, the field of computer vision has witnessed the widespread adoption of deep learning techniques, including their application in the domain of spectral reconstruction. Through the utilization of extensive training datasets, deep learning networks have demonstrated the capacity to learn the intricate mapping relationships between RGB and multispectral imagery [22]. Though convolutional neural networks (CNNs) and generative adversarial networks (GANs) have served as popular models, CNN-based approaches for spectral reconstruction face challenges such as data imbalance and computational complexity [23]. However, a noteworthy development in this arena has been the rise of Transformer models, which have achieved remarkable success in natural language processing (NLP) and have also found application in computer vision. In contrast to recurrent neural networks (RNNs) and convolutional neural networks (CNNs), Transformer models introduce self-attention mechanisms to tackle issues such as information loss and gradient vanishing [24]. Notably, the multi-head self-attention (MSA) mechanism within Transformers excels at capturing long-range dependencies and non-local self-similarity, thus mitigating the limitations associated with CNN-based spectral reconstruction algorithms.

**Figure 6:**
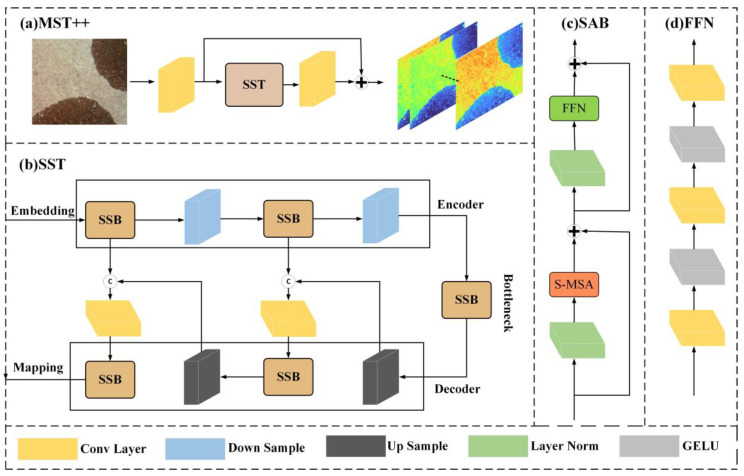
MST++ network structure diagram. (**a**) Multi-stage spectral-wise Transformer. (**b**) Single-stage spectral-wise Transformer. (**c**) Spectral-wise attention block. (**d**) Feed forward network.

Nonetheless, employing the Transformer model directly for spectral reconstruction presents certain challenges. Firstly, the multi-head self-attention (MSA) mechanism entails high computational complexity. Secondly, MSA is constrained by the input sequence length [25]. To address these issues, this study introduces the Multilevel Spectral Smart Transformer model (MST++) based on the Transformer framework [26]. The model structure is shown in Figure 6. Leveraging the sparse spatial distribution and self-similarity of multispectral image signals, the proposed approach incorporates a spectral-level self-attention mechanism (S-MSA) as a fundamental unit known as the spectral-level self-attention block (SAB). This mechanism efficiently reconstructs spectral information from RGB images. Furthermore, by integrating a spectral-level autonomous block, a single-stage spectral-level Transformer (SST) is constructed. The SST represents the input RGB image as a three-dimensional tensor and decomposes it into spectral and spatial dimensions. The structure of the SST follows a U-shaped architecture, featuring an encoder and a decoder, with both comprising multiple spectral-level self-attentive blocks (SABs), where the former extracts spectral information from the RGB image and the latter reconstructs the extracted spectral information into a multispectral image. Finally, the MST++ model is composed of multiple SSTs, working jointly to improve the spectral reconstruction process.

#### 2.3.2. Neural Network for Runway Oil Detection

Presently, RGB image-based detection stands as the dominant method for identifying oil spills on airport tarmac. Nevertheless, this method is susceptible to various factors, such as lighting conditions, color variations of the ground, viewpoint differences, and limited data, which can lead to false positives or missed detection. In contrast, the utilization of multispectral imagery offers a promising solution to overcome these challenges effectively. By capturing information beyond the visible spectrum, multispectral imaging offers enhanced discrimination and detection capabilities, thereby mitigating the aforementioned issues associated with RGB-based detection.

**Figure 7:**
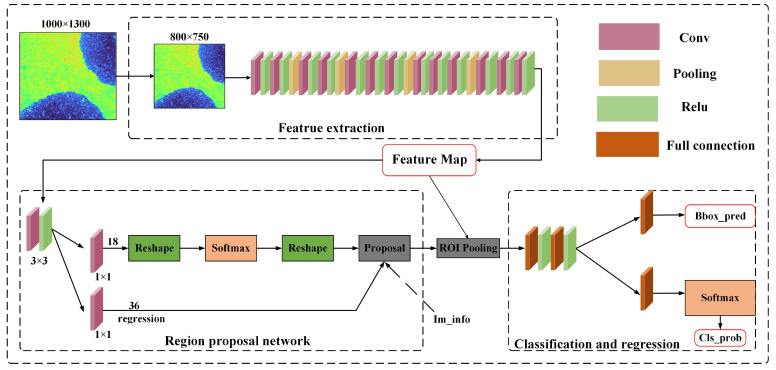
Structure of Faster RCNN network.

In this study, we employed the Faster RCNN algorithm, a deep learning-based approach, for the detection of oil spills in multispectral images of airport environments. Faster RCNN comprises four core modules: Feature Extraction Network, Region Proposal Network (RPN), Generation of Region of Interest (ROI), and Classification and Regression [27]. The Feature Extraction Network module is responsible for extracting relevant features from the input image by employing convolutional, ReLU activation, and pooling layers, which collectively capture high-level representations of the input image. The Region Proposal Network generates multiple Regions of Interest (ROIs) based on the extracted feature map. Each candidate region is assigned a probability value and is subsequently classified by a classifier. The Generation of the ROI module takes the output regions from the RPN and the feature maps from the Feature Extraction Network as input. It combines the region features from both sources and feeds them to a fully connected network for classification. The Classification and Regression module takes all the feature maps as input and outputs both the object category and its position within the image. This module employs bounding boxes to refine the object’s position, improving localization accuracy. Figure 7 illustrates the architecture of Faster RCNN, showcasing the interconnectedness of its components.

### 2.4. Model Training

In this study, MATLAB R2022a, Anaconda3 (python3.6), and PyTorch libraries were used for the implementation of the spectral reconstruction algorithms. The training and testing of the spectral reconstruction network were performed on an Intel i7-12700k CPU (3.60 GHz) (Intel Corporation, headquartered in Santa Clara, CA, USA) with 32 GB RAM (Corsair Components, Inc., based in Fremont, CA, USA) and NVIDIA RTX3080 (CUDA 11.4) (Nvidia Corporation, also located in Santa Clara, CA, USA).

The training of the spectral reconstruction network model in this study was not based on a multispectral camera for data acquisition but instead on the NTIRE 2022 Spectral Dataset [28], which consists of 1000 hyperspectral images and their corresponding RGB images and covers a wide range of scenes, such as urban, rural, and natural landscapes. The hyperspectral images had a spatial resolution of 512 × 482 and a spectral resolution of 31 bands ranging from 400 to 700 nm. The RGB images were simulated from the hyperspectral images by using the spectral response functions of a standard digital camera.

To ensure a robust evaluation of the model’s performance and generalization ability, we randomly split the dataset into a training set and a validation set in a ratio of 9:1, which allowed for a sufficient amount of data for model training while reserving an independent subset for model validation and parameter tuning. The random splitting process was repeated 10 times to minimize the potential bias introduced by a single split. During the training process, it was essential to normalize and rescale the RGB images to a range of [0, 1]. The Adam optimizer was employed for parameter optimization, with default values of β1 = 0 and β2 = 0.999. To control the learning rate, the cosine annealing method was implemented, initially set to 0.004. If the learning rate did not exhibit significant decay, the training process was halted. The evaluation metrics loss, MRAE, and RMSE were employed to assess the performance of the model.

In the oil detection network, the input consists of the reconstructed hyperspectral imaging (HSI) image, which is obtained as the output of the spectral reconstruction network. The HSI images were divided into a training set and a test set in a ratio of 9:1, following a similar procedure to that employed for the spectral reconstruction dataset. The choice of dataset split ratio (9:1) for both the spectral reconstruction and oil detection tasks is based on the common practice in deep learning to allocate a larger portion of data for training while reserving a smaller subset for validation and testing [29]. This ratio provides a balance between model training and evaluation, allowing for sufficient data to learn representative features while assessing the model’s performance on unseen data. The repetition of random splitting further enhances the reliability and reproducibility of the results by reducing the impact of data split variations [30]. Considering the size of the HSI image, patches of size 3 were utilized to extract local features. The initial learning rate was set to 0.003, and the cross-entropy loss function was employed to measure the discrepancy between predicted and ground-truth labels. By leveraging these settings, we aimed to employ the oil detection network to effectively identify and classify oil spills in hyperspectral images.

### 2.5. Evaluation Indicators

In this study, the mean relative error (MRAE) and root mean square error (RMSE) are selected as evaluation metrics for spectral reconstruction [31,32]. MRAE is utilized to measure the average relative error between the reconstructed spectra and the actual spectra, as defined by Equation (1). The MRAE value directly reflects the prediction error of the spectral reconstruction, and a smaller MRAE value indicates better spectral reconstruction performance [33]. On the other hand, RMSE is employed to quantify the discrepancy between the reconstructed spectra and the actual spectra, as defined by Equation (2). A smaller RMSE value indicates a smaller prediction error in spectral reconstruction, demonstrating the superior performance of the spectral reconstruction model. However, when using RMSE for model evaluation, it is important to consider whether the spectral range of the compared objects is the same. Different spectral ranges can lead to variations in the RMSE value, potentially impacting the evaluation results [34]. These evaluation indices provide valuable insights into the accuracy and effectiveness of the spectral reconstruction model in this study.

(5) $$MRAE=\frac{1}{N}\sum_{i=1}^{N}\frac{\left|I_{R}^{i}-I_{G}^{i}\right|}{I_{G}^{i}}$$

(6) $$RMSE=\sqrt{\left(\frac{1}{N}\sum_{i=1}^{N}{\left(I_{R}^{i}-I_{G}^{i}\right)}^{2}\right)}$$

In the above equation, N is the total number of image elements, and $I_{R}^{i}$ and $I_{G}^{i}$ denote the ith pixel value of the reconstructed spectral image and the real image, respectively.

## 3. Experiments and Discussions

### 3.1. Spectral Reconstruction Quality Assessment

To evaluate the effectiveness of the proposed spectral reconstruction network, MST++, in this study, we compared it with several advanced reconstruction networks, including HSCNN+, HRNET, and MIRNET, on the NTIRE 2022 Spectral Dataset. The evaluation metrics employed were loss, mean relative error (MRAE), and root mean square error (RMSE). The experimental results are presented in Table 1, showcasing the performance of different models on the test set. From the results in Table 1, it is evident that MST++ achieved a significantly lower MRAE score (0.1595) than HSCNN+ (0.1727), HRNET (0.1685), and MIRNET (0.1945), with reductions of 7.6%, 5.3%, and 18%, respectively. Additionally, the RMSE of MST++ was reported as 0.0194, exhibiting decreases of 9.7%, 4.7%, and 15.3%, respectively, compared with the other network models. These results clearly demonstrate that the MST++ model outperformed the other models in terms of accuracy, strongly supporting its superiority in achieving the best results in spectral reconstruction.

**Figure 8:**
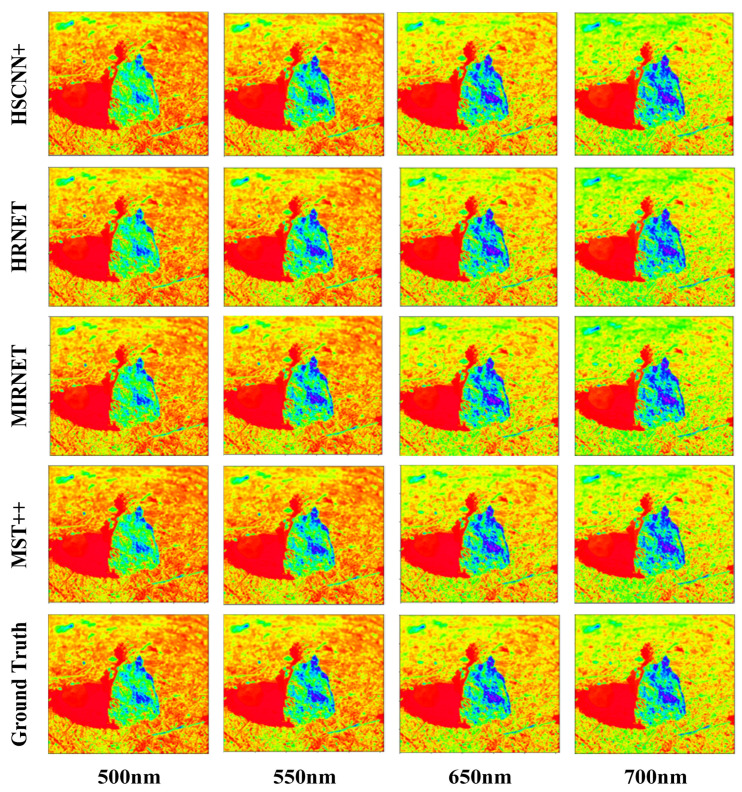
Comparison of spectral recovery of 4 bands in RGB image.

To provide a more intuitive demonstration of the different effects of each network model on spectral reconstruction and to validate the performance of MST++, we randomly selected an image from the validation set to showcase the reconstruction results. This approach ensured an unbiased evaluation of the model’s generalization ability and its effectiveness in reconstructing spectral information on images not used during training. The reconstruction results are presented in Figure 8. Specifically, four wavelengths, namely, 500 nm, 550 nm, 650 nm, and 700 nm, were chosen to display the multispectral reconstruction outcomes. These wavelengths cover the visible and near-infrared spectra, allowing for a comprehensive assessment of the model’s performance in reconstructing features across different spectral regions [35,36]. The last row shows the ground-truth spectral images for comparison and verification.

**Figure 9:**
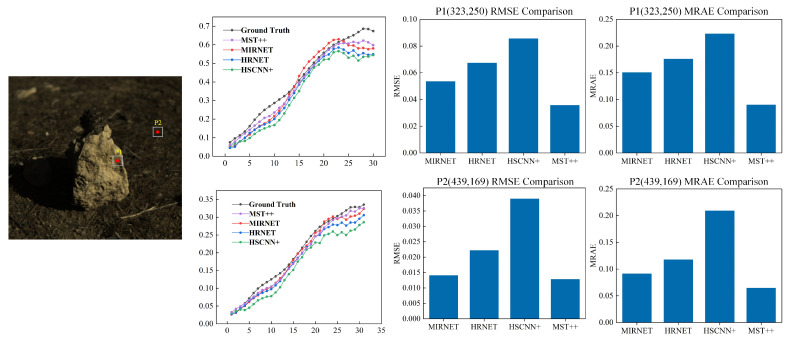
Reconstructed pixel results with coordinates p1 (323,250), p2 (439,169) and the RMSE and RMAE error histogram calculated from the model reconstruction results and the ground truth.

To further demonstrate the model’s reconstruction performance on different objects within the same image, we selected two validation points: one from the main subject area (Position 1, coordinates 323, 250) and another from the background region (Position 2, coordinates 439, 169), representing areas with distinct spectral characteristics. By randomly selecting validation points from both the subject and background for analysis, we aimed to showcase the model’s ability to reconstruct objects with varying spectral features within an image. This provides a detailed visualization of the MST++ spectral reconstruction network model’s performance in preserving the spectral information of various objects. As illustrated in Figure 9, a comparison of the pixel-level reconstruction performed by the MST++ model and three other reconstruction models for the selected image and validation points is presented. The MST++ results exhibit a closer resemblance to the ground-truth spectral images compared with the results of the other three models. This demonstrates the superior performance of MST++ in accurately reconstructing spectral information at the pixel level, both for the main subject area and the background region.

To quantitatively evaluate and clarify the superior performance of the MST++ model in pixel-level spectral reconstruction, we calculated and compared the RMSE and MRAE values of the reconstruction results obtained by different models at the selected points with respect to the ground truth, as shown in the bar chart in Figure 9. For Position 1 (main subject area), the RMSE value of the MST++ model was 0.0356, which was 0.0178, 0.0317, and 0.0499 lower than those of the MIRNET, HRNET, and HSCNN+ models, respectively. The MRAE value of the MST++ model at Position 1 was 0.0900, which was 0.0605, 0.0856, and 0.1329 lower than those of the MIRNET, HRNET, and HSCNN+ models, respectively. For Position 2 (background region), the RMSE value of the MST++ model was 0.0128, which was 0.0013, 0.0094, and 0.0262 lower than those of the MIRNET, HRNET, and HSCNN+ models, respectively. The MRAE value of the MST++ model at Position 2 was 0.0645, which was 0.0266, 0.0529, and 0.1445 lower than those of the MIRNET, HRNET, and HSCNN+ models, respectively.

These results demonstrate that the MST++ model outperformed the other three models in reconstruction performance at both positions. Moreover, the superiority of the MST++ model was more pronounced in the main subject area compared with the background region, with larger differences in RMSE and MRAE values between the other models and the MST++ model. This further validates the stronger capability and stability of the MST++ model in capturing and reconstructing the spectral features of different objects, making it suitable for integration into multispectral oil spill detection models.

**Figure 10:**
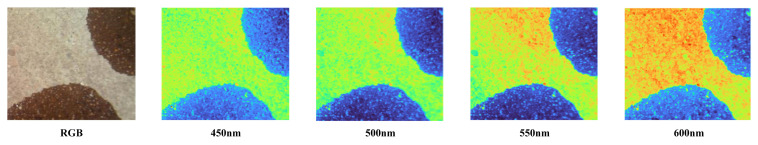
The effect of oil reconstruction.

To validate the effectiveness of the proposed spectral reconstruction network, in this study, a portion of the dataset containing oil stains on concrete pavement was utilized as input for the MST++ model. Specifically, multispectral images captured in four bands were selected for analysis, as illustrated in Figure 10. From the figure, it is evident that in the reconstructed image, the spectral details of the oil spill are effectively preserved and that the oil spill can be clearly distinguished from the road surface. This outcome highlights the capability of the MST++ model to accurately reconstruct multispectral images, making it a valuable tool for the detection of oil spills in such images. The results depicted in Figure 10 provide compelling evidence of the MST++ model’s effectiveness in capturing and reconstructing the spectral characteristics of oil spills on concrete pavement. Its ability to retain important spectral details and accurately differentiate between an oil spill and the road surface underscores its potential for efficient and reliable multispectral image detection applications.

**Figure 11:**
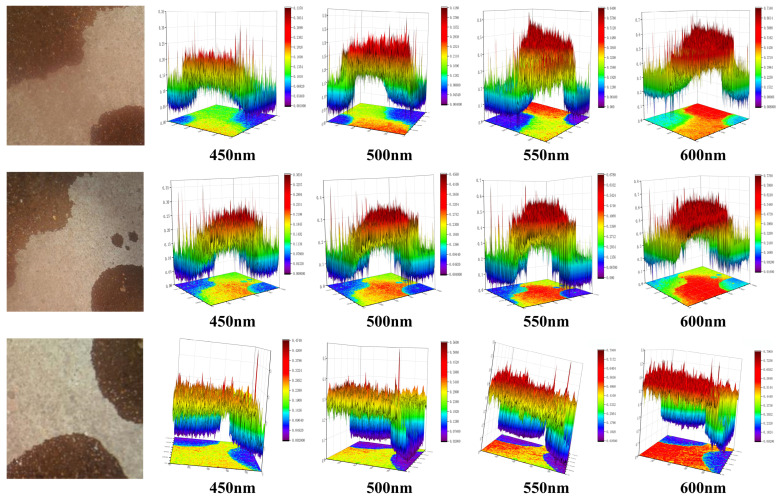
Reconstruction effect of different oil stains in different bands.

In order to verify the effects of spectral reconstruction, in this study, three images were randomly selected from the mixed-oil dataset for spectral reconstruction. Figure 11 below shows RGB images of aviation paraffin and lubricating oil, aviation paraffin and engine oil, and engine oil and lubricating oil; these were reconstructed, and the multispectral images were selected in four bands. From the figure, it can be seen that in the different bands, different types of oil spillage show clear distinctions and are clearly differentiated from the road surface. This provides a theoretical basis for the application of the proposed oil spill detection model for airport pavement monitoring.

### 3.2. Comparison of Oil Detection Models in Different Scenarios

**Figure 12:**
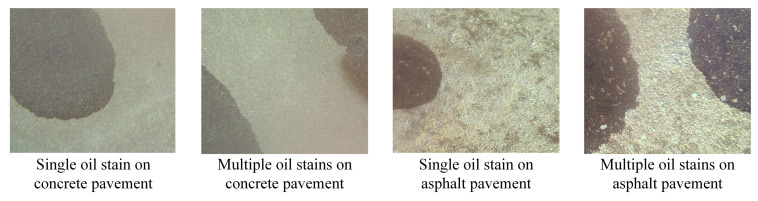
RGB images of oil in different scenes.

Based on the results of our spectral reconstruction experiments, we determined that the MST++ model is highly suitable for reconstructing spectral information of oil spills on airport runways. Therefore, in this study, we employed MST++ to transform the acquired RGB images of airport runway oil spills into HSI images. To evaluate the effectiveness of the reconstructed oil spill HSI images, we conducted detection performance tests in various airport pavement scenarios. This included scenes of single oil spills on both concrete and asphalt pavement, as well as complex oil spill scenes occurring on the same pavement. The oil spill detection model utilized in this study took the acquired RGB images and the reconstructed HSI images as inputs, generating two sets of detection results. To validate the performance of HSI image detection in oil spill detection, we designed a control test to compare these two sets of detection results. Figure 12 showcases a selection of original data without spectral reconstruction from four different scenes. These original oil spill RGB images were then inputted into the spectral reconstruction model to obtain the reconstructed HSI images of oil spillage on the airport road. Subsequently, the original oil RGB images and the reconstructed HSI images were fed to the airport road oil spill detection model, yielding the final detection results. Through this experimental setup, we aimed to evaluate the performance of the HSI image-based detection model in oil spill detection. By comparing the detection results obtained from the original oil RGB images and the reconstructed HSI images, we assessed the effectiveness of the HSI image method in accurately detecting oil spills on airport runways.

In this study, we developed an oil spill detection network model specifically designed for airport pavement. This model combines feature extraction and target classification methods to achieve accurate detection results. To ensure compatibility with the detection model, both RGB images and the reconstructed HSI images need to be resized to a dimension of 800×800 pixels. The Feature Extraction Network within the oil detection model consists of a convolutional layer, enabling the extraction of oil-related features in the four different scenes. Specifically, the features of aviation paraffin, engine oil, and lubricating oil within these scenes are extracted. Subsequently, a region candidate network is employed for classification purposes, while interest region pooling is utilized for regression analysis. To evaluate the recognition accuracy of the model, we adopted the IOU (Intersection over Union) metric, which quantifies the degree of overlap between the predicted target frame location and the actual target frame location. By assessing IOU, we can effectively measure the accuracy of a model’s predictions [37]. By combining feature extraction, target classification, and evaluation using the IOU metric, our oil spill detection network model demonstrates the ability to accurately identify and classify oil spills on airport pavement. The integration of these techniques allows for robust and reliable detection performance, supporting maintenance and safety efforts in airport environments.

**Figure 13:**
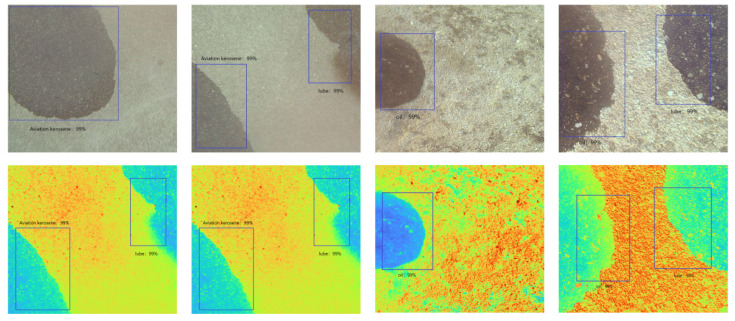
Schematic illustration of the effect of RGB and HSI image detection of oil on airport pavement in multiple scenarios.

The acquired original RGB image and the reconstructed multispectral image of the same scene were used for oil detection, and the detection results are shown in Figure 13 below.

**Figure 14:**
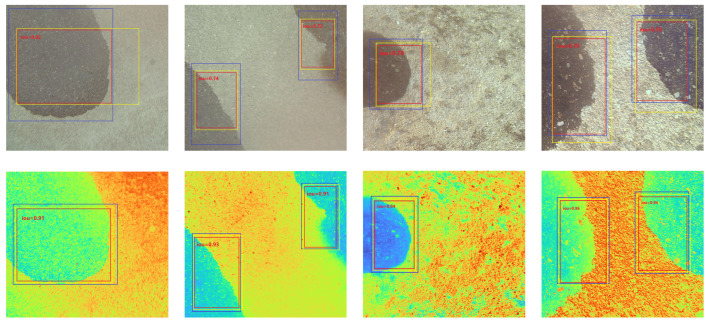
Results of oil IOUs in multiple scenarios.

By comparing the predicted oil spill locations generated by the oil spill detection model with the actual oil spill location information obtained during data annotation and calculating the Intersection over Union (IOU), we evaluated the accuracy of the model. IOU is computed as the ratio of the area of intersection between two bounding boxes to the area of their union. The results of this evaluation are presented in Figure 14, where we can observe the calculated IOU values, which indicate the degree of overlap between the predicted and actual oil spill locations. Higher IOU values indicate better alignment between the predicted and ground-truth bounding boxes, suggesting higher accuracy in detecting and localizing oil spills on airport pavement.

In Figure 14, the yellow border represents the position of the real target box, which was obtained from the labeled data, while the blue border indicates the position of the predicted target box detected by the RGB image target model. The red border represents the overlap between these two target boxes. The results presented in Figure 14 clearly demonstrate the model’s ability to accurately detect the position of oil spills in the images. The red border, representing the overlap between the predicted and real target boxes, indicates a high degree of alignment. This alignment validates the effectiveness of the detection model in precisely identifying the location of the oil spills in the images. The findings illustrated in Figure 14 provide strong evidence of the detection model’s capability to reliably detect and locate oil spills. This information is crucial to implementing appropriate measures to address and mitigate oil spills on airport pavement.

According to the IOU statistics for each of the 60 images in the prediction set of RGB and HSI images, the statistical results are shown in Table 2 below. The prediction effects of the three types of oil spills include the actual position of the oil spill, and the IOU score was over 60%. The statistics of the average IOU value are shown in the table and summarized as follows: the average IOU values of the RGB images were 0.825, 0.814, and 0.842, and the total average IOU value was 0. 827; the average IOU values of the HSI images were 0.855, 0.816, and 0.921, and the total average IOU value was 0.864. The above data show that the detection network model can be more effective in detecting oil spills in HSI images.

The level of hazard posed by different oil spill incidents on airport pavement can vary significantly. Therefore, solely relying on the detection accuracy based on IOU may not adequately reflect the effectiveness of detecting each type of oil spill. To address this, in this study, we used the detection rate and recognition rate as additional evaluation metrics. In Table 3 below, we present the detection rate, recognition rate, and detection accuracy for both the RGB images and the HSI images. These metrics aim to capture the differences in detection performance between the two types of images.

As presented above in Table 3, the detection rates for aviation paraffin, engine oil, and lubricating oil in the HSI images were 85.6%, 82.5%, and 93.2%, respectively. These rates were significantly higher than those in RGB images (aviation paraffin: 56.7%; engine oil: 54.9%; and lubricating oil: 74.0%). Moreover, the overall detection rates in the HSI images were also notably higher than those in the RGB images. These results clearly indicate the superior performance of spectrally reconstructed HSI images compared with traditional RGB images for accurately identifying and recognizing oil spills on airport pavement, suggesting that the former are better suited for the detection of oil spills in this environment, offering improved outcomes and higher detection accuracy.

In this study, we demonstrated that airport pavement oil spills can be identified by using low-cost RGB images, which can be inputted into the spectral reconstruction network proposed in this study to acquire the corresponding HSI images, which can, in turn, be used to detect the oil spills. Based on the high cost of acquiring original HSI images, the complex operation of multispectral cameras, and the varied composition of airport pavement, in this paper, we developed a better method for the detection of oil spills on airport pavement.

## 4. Conclusions

Airport runway oil spill detection plays a crucial role in ensuring aviation safety and facilitating accident investigations. Traditional RGB image-based detection methods struggle to distinguish between oil spills and water stains due to their similar color appearances in the visible spectrum. However, oil spills and water stains exhibit distinct absorption characteristics in multispectral imagery, enabling precise oil spill detection based on hyperspectral images. In this study, we established a low-cost and efficient framework for airport runway oil spill detection based on spectral reconstruction and oil spill detection.

This research study was divided into two parts. The first part focused on the spectral reconstruction of airport runway RGB images. Low-cost RGB cameras were used to quickly acquire raw data on oil spills on airport runways, which were then inputted into a spectral reconstruction network to obtain hyperspectral images of the oil spills. In the spectral reconstruction phase, we employed the MST++ spectral reconstruction network model to effectively reconstruct RGB images into hyperspectral images. Compared with other models, namely, HSCNN+, HRNET, and MIRNET, MST++ demonstrated superior performance, reducing the mean relative absolute error (MRAE) and root mean square error (RMSE) by 0.0132–0.0350 and 0.0010–0.0033, respectively, achieving excellent results of 0.1595 and 0.0194, respectively. Notably, MST++ outperformed the other three models in reconstruction quality, regardless of whether the selected coordinates corresponded to the main oil spill area or the background region. Moreover, the advantage of the MST++ model was more pronounced in the main oil spill area than in the background region. This indicates that MST++ can reconstruct hyperspectral images more accurately, thereby helping to improve accuracy in oil spill detection.

The second part of the research study focused on oil spill detection. We performed detection on the obtained hyperspectral images of oil spills by using the Fast R-CNN model. Compared with RGB images, the detection method based on hyperspectral images increased the Intersection over Union (IOU) value by 5%, while detection accuracy and completeness were improved by 25.3% and 26.5%, respectively. Successful detection was achieved in various scenarios, validating the practicality and effectiveness of the proposed method. By leveraging spectral reconstruction techniques to mine the spectral information of oil spills, the method overcomes the limitations of traditional RGB image detection, effectively improving detection accuracy. The method offers the advantages of low cost and high efficiency, presenting broad application prospects in airport operations and contributing to the enhancement of aviation safety and environmental protection. Furthermore, were the dataset further expanded and enriched, the method would have potential application value in other transportation domains and infrastructure maintenance, providing new solutions for safety monitoring and maintenance in related fields.

## Figures and Tables

**Table 1:** Spectral reconstruction evaluation indicator results.

Modeling	Loss	MRAE	RMSE
HSCNN+	0.2528	0.1727	0.0215
HRNET	0.2519	0.1685	0.0204
MIRNET	0.2484	0.1945	0.0227
MST++	0.2415	0.1595	0.0194

**Table 2:** Mean IOU statistics of airport pavement oiling HSI and RGB image prediction set.

Type of Oil Contamination on Airport Pavements	HSI Image Prediction IOU Mean	RGB Image Prediction IOU Average
Aviation kerosene	0.855	0.825
Engine oil	0.816	0.814
Lubricating oil	0.921	0.842
Aggregate	0.864	0.827

**Table 3:** Statistics of checking accuracy and completeness of the prediction set of HSI and RGB images of oil contamination on airport pavements.

Airport Pavement Grease Category	HSI Accuracy Rate	HSI Detection Rate	RGB Check Rate	RGB Detection Rate
Aviation kerosene	0.856	0.826	0.567	0.574
Engine oil	0.825	0.855	0.549	0.560
Lubricating oil	0.932	0.964	0.740	0.960

## Data Availability

The original contributions presented in the study are included in the article; further inquiries can be directed to the corresponding author.
